# Posterior reversible encephalopathy syndrome (PRES) of the isolated brainstem

**DOI:** 10.1002/ccr3.5712

**Published:** 2022-04-07

**Authors:** Yosuke Hayashi, Tomoaki Hashida, Megumi Yazaki, Tomohiko Uchida, Eizo Watanabe

**Affiliations:** ^1^ Department of Emergency and Critical Care Medicine Eastern Chiba Medical Center Togane City Japan; ^2^ Department of General Medical Science Chiba University Graduate School of Medicine Chiba Japan; ^3^ Department of Neurology Eastern Chiba Medical Center Togane City Japan

**Keywords:** brain edema, emergency medicine, hypertension, magnetic resonance imaging, neurology, pons

## Abstract

A 71‐year‐old man had disordered consciousness whose Glasgow Coma Scale was E4V1M5. His blood pressure was high, but there was no abnormality in the cerebrospinal fluid examination. The MRI finding reveals a high‐intensity area at the pons without the blood flow interruption. Thus, he has diagnosed with brainstem PRES.

## Graphical abstract

The typical MRI finding of PRES is the presence of a symmetric vasogenic edema in the parietal and occipital lobes. However, up to the 27% of cases occur at the brainstem, and it is imperative to remember this less frequent localization.

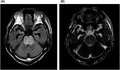

Posterior reversible encephalopathy syndrome (PRES) refers to a reversible subcortical vasogenic brain edema disorder in patients with acute neurological symptoms. The pathophysiology is mainly hyperperfusion of the brain as a sequel of hypertension.^1^

A 71‐year‐old man was taken to our hospital because of disordered consciousness. On admission, his conscious level was E4V1M5 with Glasgow Coma Scale without other neurological disorders. His blood pressure was 209/124 mmHg, but there was no abnormality in laboratory testing and cerebrospinal fluid examination. The MRI finding reveals a high‐intensity area confined at the pons with fluid‐attenuated inversion recovery imaging and apparent diffusion coefficient map (Figure 1A,B), but isointensity of diffusion‐weighted imaging and no evidence of the blood flow interruption. Thus, the patient was diagnosed with PRES of the isolated brainstem. The patient recovered with antihypertensive treatments and was discharged on the 20th day without any sequelae.

**FIGURE 1:**
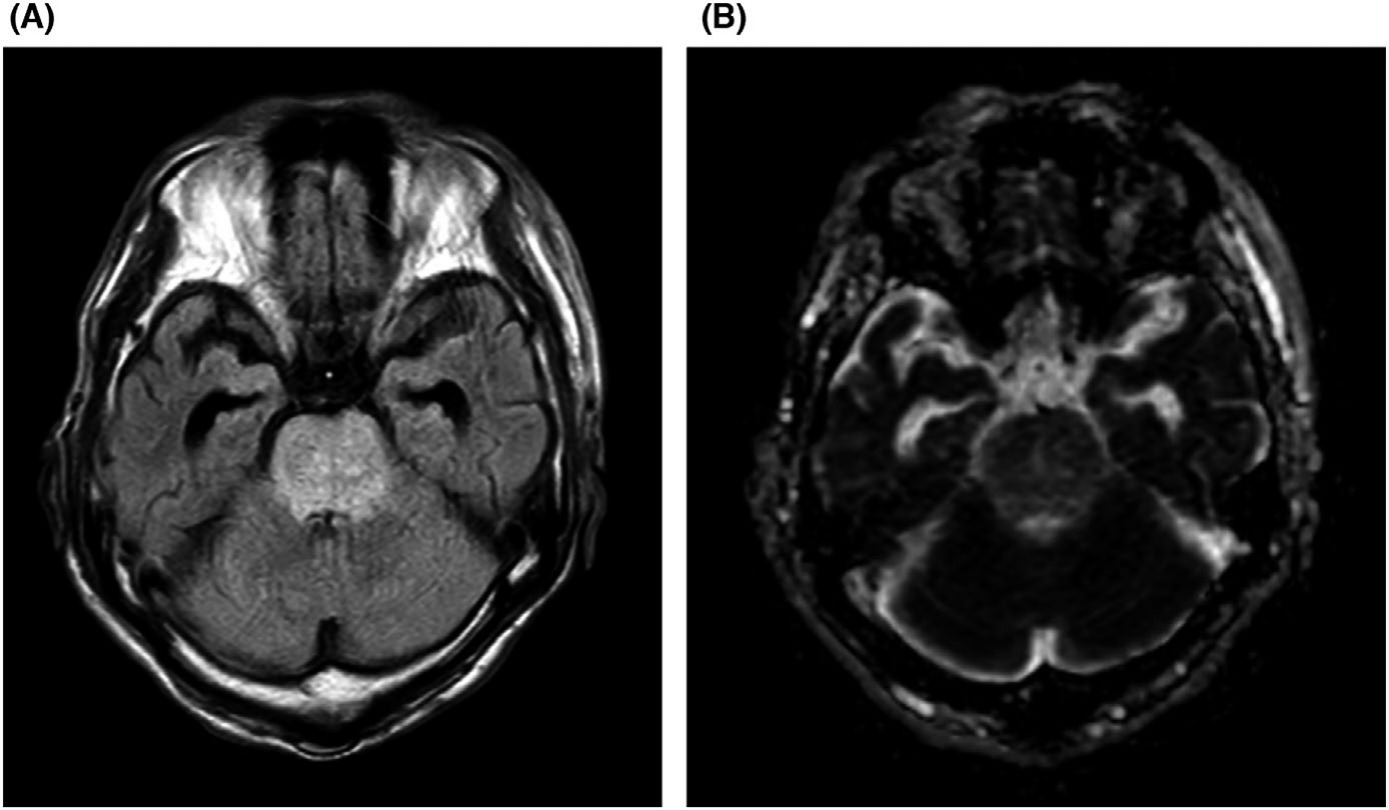
The magnetic resonance image (MRI) finding reveals a high‐intensity area at the pons with fluid‐attenuated inversion recovery (FLAIR) imaging and apparent diffusion coefficient (ADC) map (A and B), but isointensity of diffusion‐weighted imaging (DWI) and no evidence of the blood flow interruption. Both cerebellum and cerebrum were free of abnormal findings, and these findings suggest symmetric vasogenic edema at isolated pons. The characteristic of isolated brainstem PRES is that neurological symptoms are relatively mild for the abnormal imaging findings

Posterior reversible encephalopathy syndrome should be diagnosed in the setting of acute neurological symptoms in patients with renal failure, blood pressure fluctuations, autoimmune disorders, eclampsia, or under cytotoxic drugs. The typical MRI finding is the symmetric vasogenic edema in the parietal and occipital lobes. Although up to 27% of cases occur at the brainstem,^2^ it is crucial to remember this less frequent localization.

## CONFLICT OF INTEREST

None.

## AUTHOR CONTRIBUTIONS

YH, TH, MY, TU, and EW contributed to the clinical care and discussion. YH and EW wrote the paper. All authors contributed to the article and approved the submitted version.

## ETHICAL APPROVAL

None.

## CONSENT

Written informed consent was obtained from the patient's next of kin to publish this report in accordance with the journal's patient consent policy.

## Data Availability

Data sharing not applicable to this article as no datasets were generated or analyzed during the current study.

## References

[1] Fugate JE , Rabinstein AA . Posterior reversible encephalopathy syndrome: clinical and radiological manifestations, pathophysiology, and outstanding questions. Lancet Neurol. 2015;14(9):914‐925. 26184985 10.1016/S1474-4422(15)00111-8

[2] Fugate JE , Claassen DO , Cloft HJ , Kallmes DF , Kozak OS , Rabinstein AA . Posterior reversible encephalopathy syndrome: associated clinical and radiologic findings. Mayo Clin Proc. 2010;85(5):427‐432. 20435835 10.4065/mcp.2009.0590 PMC2861971

